# Determining threatened species distributions in the face of limited data: Spatial conservation prioritization for the Chinese giant salamander (*Andrias davidianus*)

**DOI:** 10.1002/ece3.3862

**Published:** 2018-02-16

**Authors:** Shu Chen, Andrew A. Cunningham, Gang Wei, Jian Yang, Zhiqiang Liang, Jie Wang, Minyao Wu, Fang Yan, Hanbin Xiao, Xavier A. Harrison, Nathalie Pettorelli, Samuel T. Turvey

**Affiliations:** ^1^ Institute of Zoology Zoological Society of London London UK; ^2^ Guiyang University Guiyang Guizhou China; ^3^ Guangxi Teachers Education University Nanning Guangxi China; ^4^ Fisheries Research Institute of Hunan Province Changsha Hunan China; ^5^ Chengdu Institute of Biology Chinese Academy of Sciences Chengdu Sichuan China; ^6^ Shaanxi Normal University Xi'an Shaanxi China; ^7^ Kunming Institute of Zoology Chinese Academy of Sciences Kunming Yunnan China; ^8^ Yangtze River Fisheries Research Institute Chinese Academy of Fisheries Science Wuhan Hubei China

**Keywords:** amphibian conservation, Cryptobranchidae, GIS, habitat suitability model, interview survey, local ecological knowledge

## Abstract

The purpose of this study was to determine whether limited occurrence data for highly threatened species can provide useful spatial information to inform conservation. The study was conducted across central and southern China. We developed a habitat suitability model for the Critically Endangered Chinese giant salamander (*Andrias davidianus*) based on one biotic and three abiotic parameters from single‐site locality records, which represent the only relevant environmental data available for this species. We then validated model quality by testing whether increased percentage of predicted suitable habitat at the county level correlated with independent data on giant salamander presence. We randomly selected 48 counties containing historical records which were distinct from, and independent of, the single‐site records used to develop the model, and 47 additional counties containing >50% predicted suitable habitat. We interviewed 2,812 respondents near potential giant salamander habitat across these counties and tested for differences in respondent giant salamander reports between counties selected using each method. Our model predicts that suitable giant salamander habitat is found widely across central and southern China, with counties containing ≥50% predicted suitable habitat distributed in 13 provinces. Counties with historical records contain significantly more predicted suitable habitat than counties without historical records. There are no statistical differences in any patterns of respondent giant salamander reports in surveyed counties selected from our model compared with the areas of known historical giant salamander distribution. A Chinese giant salamander habitat suitability model with strong predictive power can be derived from the restricted range of environmental variables associated with limited available presence‐only occurrence records, constituting a cost‐effective strategy to guide spatial allocation of conservation planning. Few reported sightings were recent, however, with most being over 20 years old, so that identification of areas of suitable habitat does not necessarily indicate continued survival of the species at these locations.

## 1 INTRODUCTION

Effective conservation management of threatened species requires a robust, evidence‐based understanding of key population parameters such as geographic distribution and habitat requirements (Segan, Bottrill, Baxter, & Possingham, 2011; Stewart, Coles, & Pullin, 2005; Sutherland, Pullin, Dolman, & Knight, 2004). However, robust data are often unavailable for extremely rare species, which are most urgently in need of management action, as the very rarity of these species can make them difficult to study or even detect using standard field survey methods (Thompson, 2013). Assessing the extent to which limited available data can provide useful insights to inform management of cryptic or poorly known threatened species therefore represents an important conservation research goal.

Habitat suitability models are a group of mechanistic statistical models widely used in ecology, which relate the frequency of species occurrences to sets of environmental variables in order to generate predictions of locations where species are expected to occur (Franklin, 2009). Conservation effectiveness remains hindered by severe funding and other social resource limitations, especially when trying to support conservation interventions for species that occur across large geographic areas (Isaac, Redding, Meredith, & Safi, 2012; Marris, 2007), and so such models can potentially constitute an important cost‐effective tool to optimize spatial prioritization of conservation activities. Considerable attention has been paid to factors that might affect the accuracy of occurrence probability and range prediction from habitat suitability models, including sample size, the use of presence–absence data versus presence‐only data, data quality and representativeness (e.g., associated with variation in habitat use with life stage), and randomness of sampling (Aranda & Lobo, 2011; Feeley & Silman, 2011; Fei & Yu, 2016; Fithian, Elith, Hastie, & Keith, 2015; Hastie & Fithian, 2013; Lütolf, Kienast, & Guisan, 2006; Zajac, Stith, Bowling, Langtimm, & Swain, 2015). In practice, such models may be forced to rely on presence‐only datasets comprising occurrence records that have been collected opportunistically rather than systematically, are of insufficient spatial resolution, and/or include bias in spatial search effort. For some threatened species, recent locality data might even be deliberately kept secret to reduce poaching risk (Meijaard & Nijman, 2014; Yang & Chan, 2016). As such incomplete and biased data often constitute the only information available for trying to determine potential geographic distributions for highly threatened species therefore, it is necessary to attempt to evaluate whether such data can provide a meaningful biogeographic signal.

**Figure 1:**
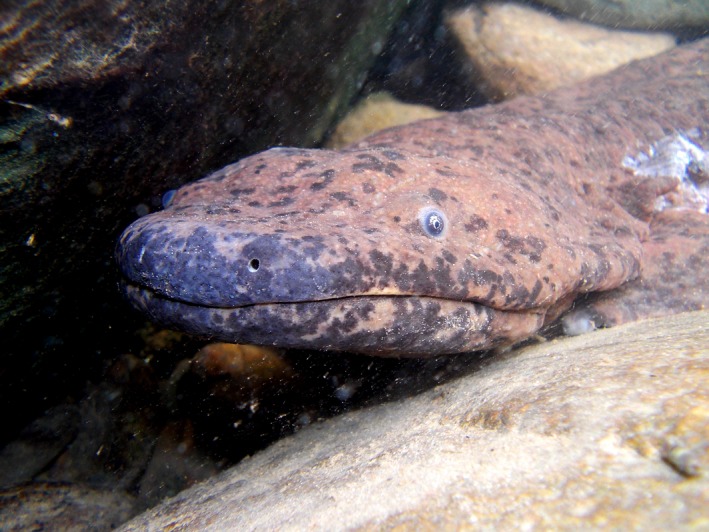
Chinese giant salamander (*Andrias davidianus*)

The Chinese giant salamander (*Andrias davidianus*) (Figure 1), the world's largest amphibian, is a cryptobranchid salamander endemic to China, where it has been historically recorded from fast‐flowing tributaries of the Yellow, Yangtze, and Pearl river systems (Fei, Hu, Ye, & Huang, 2006; Wang et al., 2004). However, the species is severely threatened both by habitat loss and by unsustainable overexploitation of wild individuals, particularly for the recently developed domestic luxury food market, and the rapidly growing giant salamander farming industry might further threaten its survival in the wild (Cunningham et al., 2016; Huang, 1982; Wang et al., 2004); the species may therefore already be extirpated from areas of suitable remaining habitat. It is listed as critically endangered by the IUCN (2016), and it is a top priority for international conservation using prioritization metrics that incorporate evolutionary history, as it is one of only three extant species in the Cryptobranchidae, a lineage that diverged from other amphibians during the Jurassic (Isaac, Redding, Meredith, & Safi, 2012). Due to severe declines observed or inferred across its range, it is now extremely difficult to detect using standard ecological survey methods (Pierson, Yan, Wang, & Papenfuss, 2014; Tapley et al., 2015), and large‐scale systematic surveys have been identified as a priority activity to inform spatial conservation planning for the species (Meredith, 2011).

Some recent field data on local giant salamander presence or absence are available at the single‐site level (Fellowes, Chan, Lau, Ng, & Siu, 2003; Pan et al., 2016; Pierson et al., 2014; Tapley et al., 2015; Wang et al., 2004), but such data are often too limited either to evaluate the continued occurrence of giant salamanders more widely across China, or to extrapolate likely habitat suitability across the species’ former range for survey planning. In addition, some recent giant salamander records might represent introduced individuals from government release programs, which may occur in unsuitable habitat outside the species’ natural geographic range and/or habitat, rather than representing surviving native populations (Cunningham et al., 2016; Zhang, Dearing, Tong, & Hughes, 2016; Zhang, Jiang, et al., 2016). A small set of single‐site giant salamander records is associated with a series of habitat parameters with local presence of the species (Table 1). Wider‐scale historical locality data, largely dating from the 1980s and 1990s shortly before the giant salamander's major population decline due to overexploitation (Cunningham et al., 2016; Wang et al., 2004), have been compiled from local gazetteer records (Fei et al., 2006). These data do not report specific localities, but are instead recorded at a relatively low spatial resolution (e.g., at the level of local municipality, county, or mountain range), with records representing relatively large areas across which key environmental parameters may show considerable variation. Analysis of these ecological and occurrence data is therefore required to assess whether they contain enough information to identify potentially suitable giant salamander habitats that could be targeted by novel field surveys.

**Table 1:** Available data on environmental variables associated with presence of Chinese giant salamander (*Andrias davidianus*), from four locations in four Chinese provinces

Province	County	Location	Mean annual temperature (°C)	Mean annual rainfall (mm)	Elevation (m)	References
Guizhou	Guiding	Yanxia	13.9	—	1,100	Li, Yu, and Ma (2009)
Henan	Lushi	Lushi County Nature Reserve	12.7	732.6	300–800	Zheng (2006)
Hunan	Dayong	Zhangjiajie	16	—	190–500	Luo, Liu, Liu, Luo, and Tang (2007)
Hunan	Dayong	Zhangjiajie	13.4–16.8	1,500	250–650	Luo, Liu, and Zhang (2009)
Hunan	Dayong	Zhangjiajie	—	—	276–470	Luo (2009)
Hunan	Dayong	Zhangjiajie (Golden Whip Stream)	12.8	1,200–1,600	491	Luo and Kang (2009)
Hunan	Sangzhi	Zhangjiajie	16	1,400	200–650	Luo, Zhang, Liu, Chen, and Gan (2009)
Shanxi	Yuanqu	Lishan National Nature Reserve	14	780	490–1,330	Guo (2011)

Thus, two limited and independent, but complementary, sources of data are available to assess the geographic distribution and habitat requirements of the Chinese giant salamander: single‐site locality records associated with specific environmental variables, and wide‐scale locality records from historical gazetteers with no associated environmental data. In order to provide an improved baseline for prioritizing field conservation activities for the Chinese giant salamander, we use the environmental data associated with single‐site locality records together with open‐source ecological data to develop the first predictive habitat suitability model for this highly threatened species. We then assess the likely accuracy of this model both using the historical gazetteer record as an independent comparative data source and by ground‐truthing model predictions with data from a new large‐scale questionnaire survey conducted across central and southern China, to determine the extent to which incomplete occurrence data for highly threatened species can provide useful information on their geographic distribution.

## 2 METHODS

### 2.1 Habitat suitability model

Eight studies documenting environmental data associated with Chinese giant salamander locality records are available in the Chinese literature, from four provinces (Hunan, *n* = 5; Guizhou, *n* = 1; Henan, *n* = 1; Shanxi, *n* = 1) (Table 1). In addition to specific water quality or microhabitat parameters for which country‐level spatial mapping data are unavailable (e.g., flow rate, dissolved oxygen, nitrogen, water hardness, substrate, bank gradient), these locality records provide site‐specific information on elevation, mean annual temperature, and mean annual precipitation. We included these three predictor variables in our habitat suitability model, using the following ranges from the literature to define suitable giant salamander habitat: 190–1,330 m a.s.l. elevation, 12.7–16.8°C mean annual temperature, ≥732.6 mm mean annual precipitation (Table 1). We also included vegetation cover as a fourth predictor variable, as available evidence suggests that Chinese giant salamanders require extensive bankside vegetation and do not occur in extensively human‐modified landscapes such as cropland, bare ground, or urban environments, unlike Japanese giant salamanders (*Andrias japonicus*) (Browne et al., 2014).

We carried out spatial analyses using ArcGIS 10.1 (ESRI, 2014). We downloaded maps of Chinese administrative areas, elevation, and land use from DIVA‐GIS (available at http://www.diva-gis.org/Data); we extracted the three vegetation categories of tree cover, shrub cover, and tree cover/other natural vegetation mosaic from the land‐use map and grouped them into a single forest cover category for analysis. This coarse scale was used due to a lack of more detailed habitat data for CGS, other than an association with vegetation cover (Table 1). We downloaded mean monthly temperature and precipitation data from the WorldClim global climate database (available at http://www.worldclim.org/current) at a resolution of 30 arc seconds and averaged these data to generate measures of mean annual temperature and precipitation. We produced a habitat suitability model for the Chinese giant salamander based on predicted habitat suitability for China using the Raster Calculator in the Spatial Analyst Tools in ArcMap 10.1 by intersecting the selected ranges of the four environmental predictor variables. We calculated the percentage of suitable giant salamander habitat in each Chinese county (*n* = 2,852) using the Zonal Statistics in the Spatial Analyst Tools in ArcMap 10.1.

All Chinese counties with historical giant salamander gazetteer records listed in Fei et al. (2006) were given the value 1 (presence). Some historical gazetteer localities were recorded at the coarser resolution of municipality or mountain area; in these cases, all counties belonging to the relevant administrative or geographic areas were categorized as 1. All other counties lacking historical gazetteer records were given the value 0 (absence). We used these presence/absence values to produce a historical distribution map for the Chinese giant salamander using ArcMap 10.1.

We used a randomization approach to test the significance of the observed relationship between historical giant salamander records and the proportion of predicted suitable salamander habitat. We did not logit‐transform the proportion data as they contained a high frequency of zeroes. At each iteration, for a total of 10,000 iterations, we randomly resampled the binary indicator variable of historical giant salamander presence and fitted a linear model specifying the resampled habitat variable as a predictor of proportion of suitable habitat. We stored the *F* statistic from each of these models to derive a null distribution of effects. We calculated a two‐tailed *p* value as the proportion of iterations yielding *F* statistics greater than the true *F* calculated from a linear model fitted to the raw data.

### 2.2 Study area

We tested model predictions as part of a large‐scale survey to assess the current status of giant salamanders across China (see Cunningham & Chen, 2016 for details of all fieldwork activities). We randomly selected 50 counties containing historical giant salamander records and 50 further counties from a sample of all counties that contained >50% predicted suitable giant salamander habitat and which did not have a historical record (using the RANDBETWEEN function in Microsoft Excel v. 14.4.8). We were able to conduct interview surveys in 95 of the selected 100 target counties (48 counties containing historical records and 47 counties containing >50% predicted suitable habitat). These sites represent 15 Chinese provinces or equivalent administrative units (Anhui = 6, Chongqing = 3, Fujian = 1, Gansu = 4, Guangdong = 1, Guangxi = 10, Guizhou = 32, Henan = 3, Hubei = 4, Hunan = 12, Jiangxi = 2, Shaanxi = 3, Sichuan = 9, Yunnan = 1, Zhejiang = 4). As the cost and effort of surveying sites were high and associated logistics were challenging, it was impractical to survey additional sites where we did not think giant salamanders were likely to occur.

### 2.3 Questionnaire survey

The use of community‐based interview surveys has recently been shown to constitute an effective survey method for detecting Chinese giant salamanders (Pan et al., 2016), and so fieldwork to investigate giant salamander status was carried out by conducting interviews in villages within 1 km of a 1‐km target stretch of potentially suitable giant salamander habitat in each selected county (fast‐flowing rocky tributaries within or adjacent to forest; Browne et al., 2014; Fei et al., 2006) as identified by county‐level fisheries and/or forestry bureau officials. We aimed to conduct 30 interviews per county, either as 10 interviews each in three villages or more interviews in fewer villages, depending on the number of communities available for sampling within the survey region; a minimum target number of 10 interviews per village will likely capture most or all existing variation in relevant experiences for many respondent groups (Guest, 2006). Respondent selection criteria/methods and interview protocols are given in Pan et al. (2016). Project design was approved by the Zoological Society of London's Ethics Committee (ref. WLE569).

We used a standard questionnaire for all interviews, which took *c*. 20–30 min to complete, and which contained a series of descriptive, contrast, and structured questions (Appendix S1). Following an initial study around three national nature reserves in Guizhou in 2013 to trial interview methods (Pan et al., 2016), we conducted interviews between May 2013 and June 2016. Interviews were conducted and recorded in Chinese by Chinese field teams led by the authors and who received training in standardized interview techniques before fieldwork commenced. As part of a wider series of questions, we asked respondents whether they knew what giant salamanders were, to describe their appearance and to identify, without any prompting, the giant salamander from illustrations of a range of salamander species found in China, taken from Fei, Hu, Ye, & Huang (2006). If respondents could correctly identify and describe the Chinese giant salamander, we asked them whether they had seen the species, and if so how recently. Respondents reported sighting records using a variety of different methods for describing the timing of past events, and we converted alternative formats to direct calendar years for analysis using the approach described by Turvey et al. (2016). As interviews were conducted across a period of 3 years due to the logistical demands of fieldwork, we then converted sighting records to number of years before the date on which interviews were conducted, to allow comparison between sites.

We investigated differences in the pattern of respondent reports of giant salamanders between surveyed counties containing historical giant salamander records and surveyed counties selected from our habitat suitability model using R version 3.4.1 (R Core Team, 2017). Differences in the overall proportion of each county type (historical record vs. habitat suitability) from which we obtained giant salamander sighting reports were investigated using chi‐squared tests. We investigated other potential effects of county selection method (0 for selection using habitat suitability model, or 1 for selection based on the existence of historical giant salamander records) on respondent reports of giant salamanders across the surveyed counties using mixed effects models, as we wanted to include the Chinese province in which each county is located as a random effect on the model intercept to control for potential variation in survey effort (as different provinces were investigated by different Chinese survey teams). We used our observed data structure (replication of records within and among grouping levels of random effects) to investigate the probability of detecting a true difference between our two datasets (historical records vs habitat suitability) given our baseline probability in the reference category of model history, and a range of true differences in reporting probabilities (Appendix S2).

We investigated differences in proportions of respondents reporting giant salamander sightings per county by fitting a binomial mixed effects model using the “glmer” function in the R package “lme4” (Bates, Maechler, Bolker, & Walker, 2015), with county selection method as a fixed effect and province as a random intercept. We calculated overdispersion for this model following guidelines in Harrison (2014). We detected strong overdispersion, with model residuals demonstrating approximately 10 times the expected variance, so we fitted an observation‐level random effect (OLRE) to control for overdispersion following Harrison (2015). The OLRE model significantly improved model fit relative to the overdispersed model ($\chi_{1}^{2}$ = 759.86, *p *<* *.001). We investigated differences in time (years) since the most recently reported giant salamander sighting per county by fitting a Poisson mixed effects model using the “glmer” function in the R package “lme4” (Bates et al., 2015), with county selection method as a fixed effect and province as a random intercept. As with the proportion models, this model exhibited strong overdispersion (variance inflation factor = 10.42), and so we refitted the model with a negative binomial error structure using the glmmADMB package (Fournier et al., 2012; Skaug, Fournier, Bolker, Magnusson, & Nielsen, 2016). The negative binomial model resulted in a significant improvement in fit over the Poisson model ($\chi_{1}^{2}$ = 375.80, *p *<* *.001). Finally, we investigated differences in mean time (years) since all respondents reported giant salamander sightings per county. As this response contained a mix of zeroes and noninteger values, we fitted a mixed effects model with a Tweedie error structure using the “cpglmm” function in the R package “cplm” (Zhang, 2013). Unlike Poisson count models, Tweedie models automatically model overdispersion in the data by estimating a dispersion parameter. As for the previous models, the Tweedie model contained county selection method as a fixed effect and a random intercept for province. We derived *p* values from all mixed effects models by comparison of nested models using a likelihood ratio test.

## 3 RESULTS

**Figure 2:**
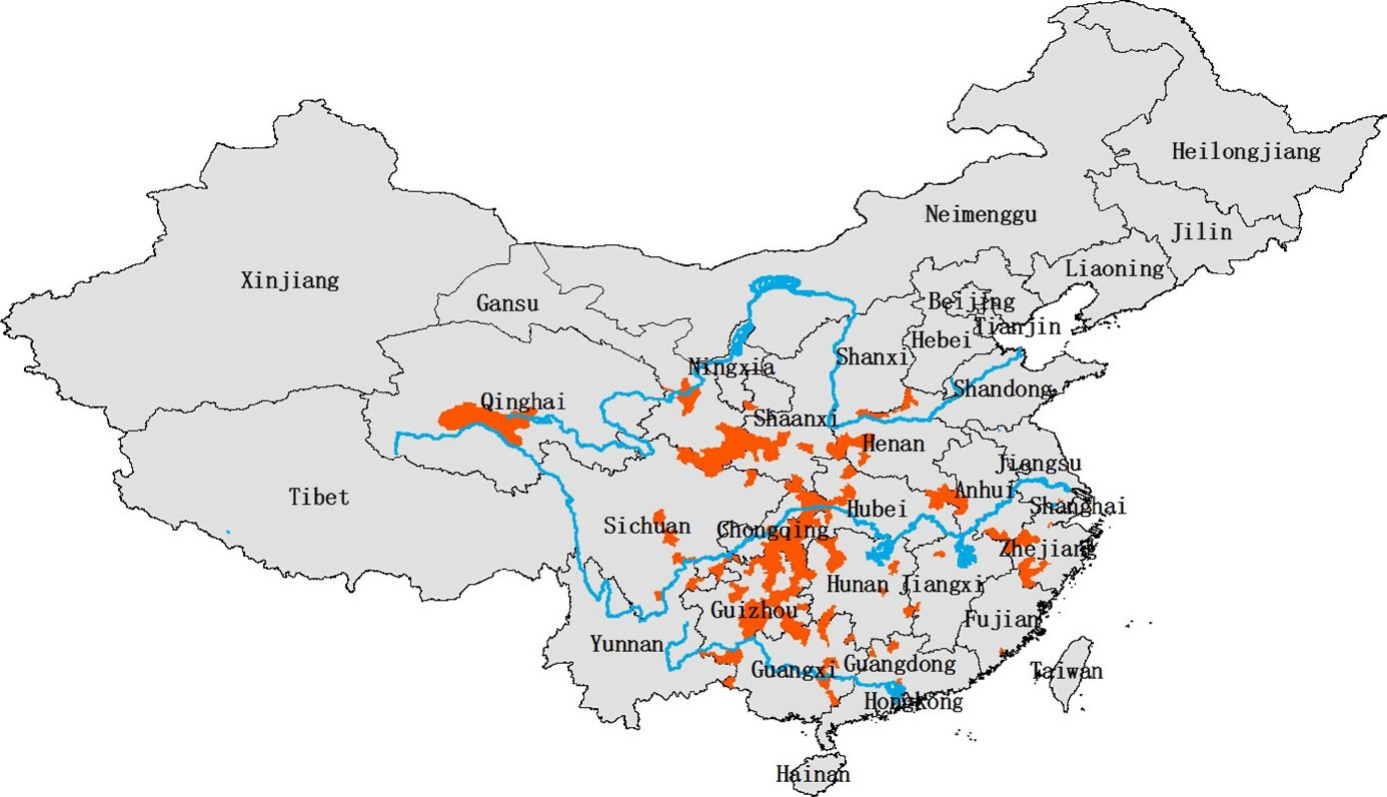
Distribution (red) of counties containing historical gazetteer records of Chinese giant salamander (*Andrias davidianus*). Data from Fei et al. (2006)

Historical giant salamander gazetteer records are documented from 145 counties (5.1% of the total number of Chinese counties) across 18 Chinese provinces or equivalent administrative areas (Anhui, Chongqing, Fujian, Gansu, Guangdong, Guangxi, Guizhou, Henan, Hubei, Hunan, Jiangsu, Jiangxi, Qinghai, Shaanxi, Shanxi, Sichuan, Yunnan, Zhejiang) (Figure 2). Guizhou has the highest number of gazetteer records (30 counties), followed by Gansu (13 counties), Guangxi (12 counties), Henan (12 counties), Hubei (11 counties), Sichuan (11 counties), and Hunan (9 counties).

**Figure 3:**
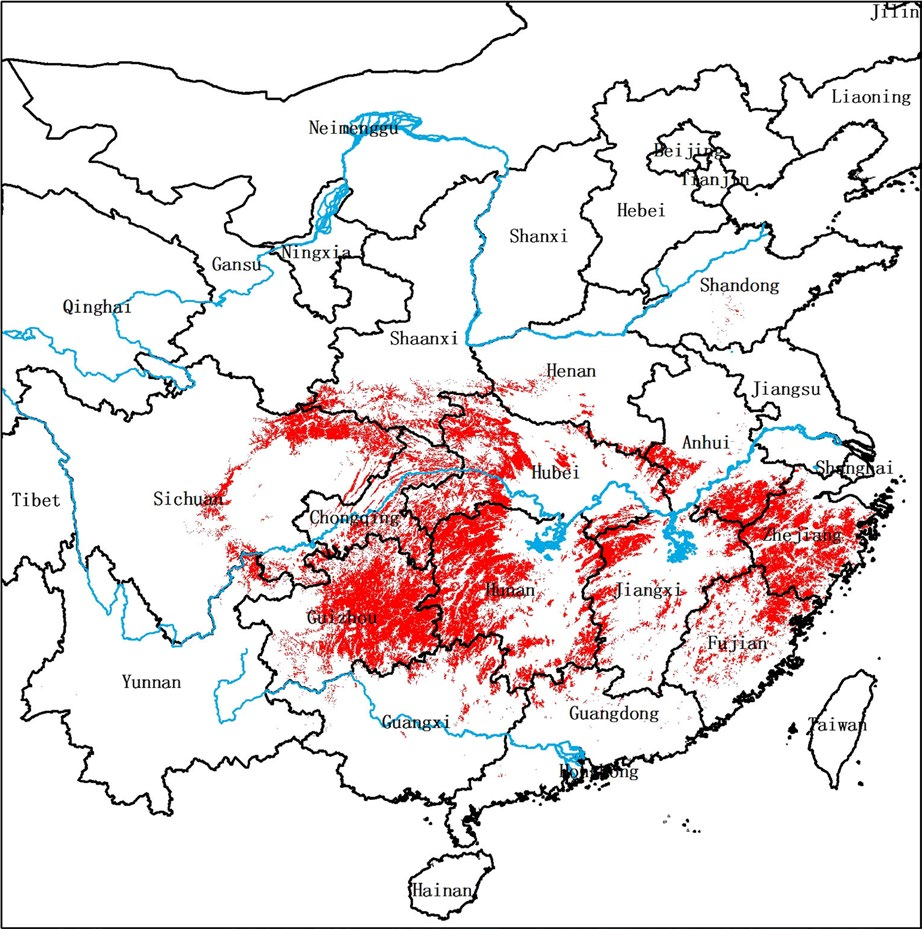
Distribution (red) of suitable Chinese giant salamander (*Andrias davidianus*) habitat across China according to the output of our habitat suitability model

Our habitat suitability model predicts that suitable habitat for the Chinese giant salamander is found widely across central and southern China (Figure 3). The percentage of predicted suitable giant salamander habitat present in different Chinese counties across the entire country ranges from 0% to 95.7%. Counties with ≥50% predicted suitable habitat (*n* = 156, representing 5.5% of the total number of Chinese counties) are distributed in 13 provinces or equivalent administrative areas (Anhui, Chongqing, Fujian, Guangxi, Guizhou, Henan, Hubei, Hunan, Jiangxi, Shaanxi, Sichuan, Yunnan, Zhejiang). Almost half of these counties are in Guizhou (*n* = 44, including 22 counties with ≥80% suitable habitat) and Hunan (*n* = 32, including 7 counties with ≥80% suitable habitat); other counties with ≥50% suitable habitat are mainly in Zhejiang (*n* = 25), Hubei (*n* = 14), Anhui (*n* = 12), and Sichuan (*n* = 8).

Predicted suitable habitats are present in 116 of the 145 counties (80.0%) that contain historical giant salamander gazetteer records. The percentage of predicted suitable habitat in these 116 counties ranges from <1% to 95.5%, with 51 containing ≥50% predicted suitable habitat; these 51 high‐suitability counties are mainly distributed in Guizhou (*n* = 23), Zhejiang (*n* = 7), and Hunan (*n* = 6). Counties with historical giant salamander gazetteer records contain a significantly higher percentage of predicted suitable giant salamander habitat (34.6%) compared to counties without historical records (6.1%) (permutation test *p*_RAND_ < .001).

**Figure 4:**
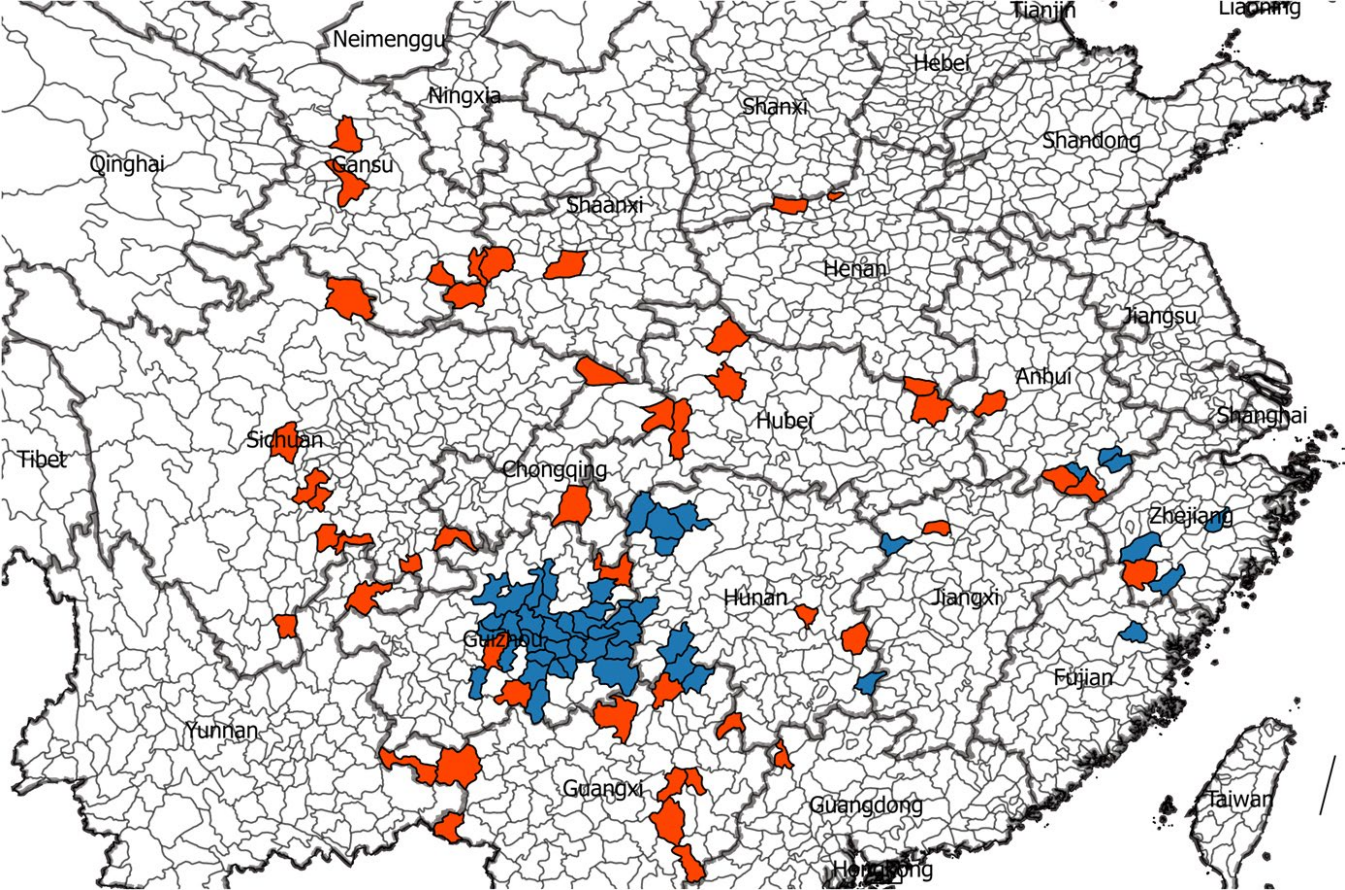
Distribution across China of surveyed counties containing historical giant salamander records (red) and surveyed counties selected from our habitat suitability model (blue)

We interviewed 2,812 respondents in 95 counties (mean age = 47.11, age range = 15–89, *SD* = 14.56; male = 69.6%, female = 30.4%; mean number of interviews/county = 29.6, range = 11–36, *SD* = 2.57) (Figure 4). In total, 1,299 respondents (46.2%) reported having seen wild giant salamanders, with 1,146 respondents (40.8%) providing a last‐sighting date (Table S1). There were no statistical differences between surveyed counties containing historical giant salamander records and surveyed counties selected from our habitat suitability model, either in the overall proportion of counties from which we obtained giant salamander sighting reports (historical: 43/48; model: 40/47; χ^2^ = 0.12, *df* = 1, *p *=* *.73), in the proportion of respondents who had seen giant salamanders (historical: mean = 0.47; model: mean = 0.45; $\chi_{1}^{2}$ = 2.37, *p *=* *.12), in time (years ago) since the most recent reported giant salamander last‐sighting date/county (historical: mean = 5.37 years ago; model: mean = 8.03 years ago; $\chi_{1}^{2}$ = 1.62, *p *=* *.20), or in time (years ago) since mean giant salamander last‐sighting date/county (historical: mean = 20.59 years ago; model: mean = 20.72 years ago; $\chi_{1}^{2}$ = 0.002, *p *=* *.96). Our analysis of statistical power revealed that for our data structure, we would have >80% power to detect a difference of 10% or more in reporting probabilities (Appendix S2).

## 4 DISCUSSION

The Chinese giant salamander is one of a large number of highly threatened but poorly known species for which only relatively limited and geographically unevenly sampled, presence‐only ecological data are available to inform conservation assessment and management. However, we demonstrate that the restricted available ecological knowledge‐base for the Chinese giant salamander is still sufficient to develop a habitat suitability model that shows close spatial congruence with independently derived historical distribution data for the species, and predicts occurrence of the species at geographical localities where local observers report statistically similar levels and patterns of giant salamander encounters compared to areas of known giant salamander distribution in China. These two independent approaches for assessing and validating the quality of our habitat suitability model demonstrate that even data for a limited series of environmental parameters can provide a robust baseline on species ecology that can be used to understand likely geographic distributions and to guide spatial allocation of conservation resources and planning.

Field validation can sometimes reveal poor performance of habitat suitability models, even when numerous parameters are used to populate the model (e.g., Anderson et al., 2016). Our predictive model was only able to include data for four environmental parameters associated with giant salamander presence. These comprised three abiotic parameters (elevation, mean annual temperature, mean annual precipitation) and one biotic parameter (vegetation cover), all of which have previously been demonstrated to constitute important predictors of distribution for amphibian species (Buckley & Jetz, 2007; Chen, 2013). Other environmental parameters, such as fish presence, water depth, and speed of water flow, also have been found to be predictors of the distribution of some amphibian species (Manenti & Pennati, 2016). Unfortunately, information about how these (or other) parameters affect the distribution of CGS is unknown, so they could not be included in our predictive model. Attempts to develop habitat suitability models for other poorly known species with limited associated ecological data should assess which environmental factors are known to limit the distribution of better‐studied related species, as exclusion of such parameters would be expected to reduce model performance.

Although Chinese counties with historical giant salamander records contain a significantly higher percentage of predicted suitable giant salamander habitat compared to counties without historical records, the congruence between counties identified by our predictive habitat suitability model and by our descriptive historical species distribution model is not complete. In addition to the possibility for error introduced by using only a small number of environmental parameters, and/or a potentially incomplete understanding of variation in giant salamander environmental tolerance associated with these parameters, this incomplete spatial agreement in inferred giant salamander distribution across China might be caused by several additional factors. Counties selected on the basis of habitat suitability might lack existing salamander records due to incomplete past survey effort across the large historical geographic distribution of this species, with many local populations potentially scientifically undetected or unreported in areas of suitable habitat within their range (Edwards, Cutler, Zimmermann, Geiser, & Alegria, 2005; Engler, Guisan, & Rechsteiner, 2004; Guisan et al., 2006). Indeed, our large‐scale questionnaire survey provides indirect evidence, based on local reports, of recent or past giant salamander occurrence in 42 Chinese counties for which no historical records are known to exist, but for which likely giant salamander occurrence was predicted by our model. Habitat suitability models have successfully detected previously unknown populations of many other rare, cryptic, or otherwise poorly studied species (Cleve, Perrine, Holzman, & Hines, 2011; Ferreira de Siqueira, Durigan, de Marco Júnior, & Peterson, 2009; Menon, Choudhury, Khan, & Peterson, 2010; Raxworthy et al., 2003; Rebelo & Jones, 2010).

Counties with historical giant salamander records might not have been identified on the basis of habitat suitability for a variety of reasons. As some historical gazetteer localities were only recorded at the coarse resolution of municipality or mountain area, it is possible that some counties included within these broader administrative or geographic areas, which were interpreted as having contained giant salamanders in our historical dataset, might not actually contain suitable environmental conditions for salamanders, and might never have been home to wild populations. In addition, China has experienced extensive habitat loss in recent decades, and national afforestation and reforestation statistics mask the ongoing degradation of native forest biodiversity, including within protected areas (Hua et al., 2016; Zhang & Song, 2006; Zhang et al., 2010). It is therefore likely that suitable vegetation cover has been lost from some historical giant salamander localities, leading to their exclusion from our predictive habitat suitability model based on recent DIVA‐GIS land‐use data.

Furthermore, although giant salamander populations across China are currently interpreted as conspecific, considerable genetic variation and phylogeographic structuring have been detected between populations occupying different river drainages (Murphy, Fu, Upton, De Lama, & Zhao, 2000). Environmental data associated with giant salamander presence are available from only four provinces, with nearly all available data from Hunan (Table 1), and it is possible that this geographically restricted baseline fails to capture true levels of variation in environmental tolerances shown by different giant salamander populations across China. Indeed, our model notably fails to predict the potential occurrence of giant salamanders on the high‐elevation Qinghai–Tibet Plateau. A giant salamander specimen was reportedly collected in 1966 from the headwaters of the Yangtze River in Qumalai County, Qinghai Province (Figure 2), potentially representing a disjunct, isolated salamander population occurring at an elevation >2,000 m higher than any other known population (Chen, 2011; Pierson et al., 2014). The existence of this population has not been confirmed (Pierson et al., 2014), and we were unable to include data for this record in our predictive model due to uncertainty over its exact provenance or associated environmental conditions. If this constitutes a true giant salamander population, it is likely to be genetically and ecophenotypically distinct with different patterns of environmental tolerance to lower‐elevation giant salamander populations and could even represent a cryptic species.

Despite this minor variation in historical versus predicted salamander distribution across China, the general accuracy of our habitat suitability model is supported by the close statistical congruence shown by the pattern and timing of giant salamander reports made by local respondents across surveyed counties irrespective of which method was used for county selection, indicating that regions identified using our predictive model show a similar signal of giant salamander detectability based on community‐based interviews as regions where giant salamanders are reported to have occurred in the past. Interview data collected from untrained local respondents do not represent direct observations of a target species made by scientific experts and therefore include the potential for both error and bias when inferring species presence (McKelvey, Aubry, & Schwartz, 2008). However, we consider it highly unlikely that this statistical congruence represents an artefactual “false‐positive” signal, as the Chinese giant salamander has cultural and economic importance in China (Cunningham et al., 2016; Pan et al., 2016) and our interview design aimed to minimize the potential for inaccuracy by requiring respondents to identify and describe the species correctly. Other community‐based interview surveys conducted in China have shown that patterns of local ecological knowledge on the local status of charismatic freshwater vertebrates and other rarely encountered species match independently derived scientific field data on spatiotemporal population trends for these taxa (Turvey et al., 2013, 2016).

We note, however, that respondent experience of past giant salamander sightings, supporting our prediction of local habitat suitability, does not necessarily indicate continued survival of the species across the survey region, as very few reported sightings had been made within the past decade, and most were over 20 years old. While our predictive habitat suitability model is therefore a robust indicator of former salamander occurrence, intensive overexploitation of giant salamander populations has recently occurred across China (Cunningham et al., 2016; Wang et al., 2004). Our large dataset of respondent reports of past giant salamander sightings made during recent decades therefore cannot be used to confirm the continued occurrence of the species anywhere across its range. Giant salamanders are vulnerable both to overexploitation and to habitat destruction through loss of riparian vegetation cover (from agricultural conversion and urbanization) and aquatic habitats (from water development projects and pollution), which have modified Chinese natural landscapes dramatically and present substantial challenges for future conservation of giant salamanders and many other species (Zhang, Dearing, et al., 2016; Zhang, Jiang, et al., 2016). However, the absence of recent giant salamander reports from remaining areas of suitable habitat as revealed by this study suggests that overexploitation is likely to be a more serious threat to the species. Any surviving giant salamander populations across our study area are clearly at high risk of continued exploitation; however, reporting the outputs of our habitat suitability model for the species at the broad country‐wide scale presented here is unlikely to pose an additional threat.

Our assessment of the information content associated with ecological data available for the Chinese giant salamander reveals that even a restricted range of environmental correlates derived from a limited sample of presence‐only occurrence records can, at least in some cases, be used to develop robust models with strong predictive power. In the case of the Chinese giant salamander, this improved understanding of the likely distribution of suitable habitats can be used further to investigate continued survival of local populations in high‐suitability sites, especially at sites where local respondents have reported more recent giant salamander sightings, to assess habitat suitability within existing protected areas that have already been established for the species, and to inform site selection for other conservation activities such as reintroduction and restocking (Cunningham et al., 2016; Zhang, Dearing, et al., 2016; Zhang, Jiang, et al., 2016). A larger series of static and dynamic environmental variables would undoubtedly refine our habitat suitability model, and confirmation of continued salamander existence across areas of high habitat suitability requires additional direct field investigation and systematic collection of presence–absence data. However, available independent spatial and survey data indicate that even the simple model we have developed statistically matches independent available data and accurately describes the species’ recent geographic distribution. Our study therefore supports the potential applicability of similarly limited occurrence data for setting cost‐effective yet meaningful conservation baselines for other poorly known threatened species, and for evaluating potential responses to future environmental and climatic change (e.g., Duan, Kong, Huang, Varela, & Ji, 2016). The modern conservation toolkit will have to draw upon different complementary and often limited, incomplete, or biased types of data in order to prevent future extinctions of highly threatened species in China and elsewhere. Realistically, we have no choice but to utilize whatever information is available on such species, and to continue to develop approaches to critically assess the extent to which imperfect data are useful and can be used to inform conservation planning (Hirzel, Le Lay, Helfer, Randin, & Guisan, 2006; Zajac et al., 2015).

## AUTHOR CONTRIBUTIONS

A.A.C., S.T.T., and S.C. conceived the ideas; A.A.C. obtained the funding; S.C., G.W., J.Y., Z.L., J.W., M.W., F.Y., and H.X. collected the data; S.C., S.T.T., X.H., and N.P. analyzed the data; and S.T.T. and S.C. led the writing; all authors contributed to the writing of the manuscript.

## CONFLICT OF INTEREST

None declared.
