# Supplementary material for: Determining threatened species distributions in the face of limited data: Spatial conservation prioritization for the Chinese giant salamander (Andrias davidianus)
Source: Ecol Evol. 2018 Feb 16;8(6):3098–108. doi: 10.1002/ece3.3862 (PMC5869214; doi:10.1002/ece3.3862)
Supplement: Supplementary file 1 [file ECE3-8-3098-s001.docx]

**SUPPLEMENTARY INFORMATION**

**Determining threatened species distributions in the face of limited data: spatial conservation prioritization for the Chinese giant salamander (*Andrias davidianus*)**

Shu Chen^1^, Andrew A. Cunningham^1,^*, Gang Wei^2^, Jian Yang^3^, Zhiqiang Liang^4^, Jie Wang^5^, Minyao Wu^6^, Fang Yan^7^, Hanbin Xiao^8^, Xavier A. Harrison^1^, Nathalie Pettorelli^1^ and Samuel T. Turvey^1,^*

**Appendix S1 - Villager Questionnaire**

Interview number:

Location of interview:

Date:

Name of interviewer:

**Opening statement:** “We are local scientists from ……………….., conducting ecological/environmental research. We are conducting a study on the animals and aquatic resources found in this region, and would like to ask you some questions for our studies, because you know a lot more about the environment here than we do. We have a questionnaire that takes about 20 minutes to complete. All information that you tell us will be completely confidential, we will not write down your name, and you will not be identified – we just want to try to learn more about the local environment from you.”

**A. BACKGROUND QUESTIONS**

1. Are you prepared to participate in this survey and answer the following questions? Y/N
2. Male/Female
3. Age:
4. Ethnicity:
5. How many years have you lived in this local region?
6. Occupation:

**B. CGS QUESTIONS**

1. Do you know what a CGS is? Y/N

If Y: ask informant to describe the species (emphasizing its size)

If N: prompt the informant with a CGS photograph

(show series of photographs of aquatic species, and ask informant to identify CGS accurately)

1. Are there any local names for the CGS?
2. Are there any stories, legends, myths, traditions or customs about CGS?
3. Have you ever seen a *wild* CGS around here? Y/N/don’t know

If Y:

- Describe the last time you saw a *wild* CGS (specific location, year, time of year if known)
- How many times have you seen a *wild* CGS? Describe all previous sightings (year and location)

*** Try to mark each locality on a high-resolution map as accurately as possible (try to record approximate co-ordinates, distance from community, and name of river drainage/watershed)*

*** When recording sighting dates, specifically report whether informants stated a calendar year (e.g. 1993), or whether they said “XX years/decades ago” or “in the 1980s/1990s etc.”. Try to encourage the informant to give a direct calendar year if possible.*

If N:

- Do you know anyone else in this community who has seen a *wild* CGS? If Y, where/when?

1. Do people ever eat *wild* CGS in this community?

- Yes (today)
- Yes (but only in the past)
- Never
- Don’t know

1. Have you ever eaten *wild* CGS? Y/N/don’t know

If Y:

- When was the last time you ate *wild* CGS?
- How many times per year do you eat *wild* CGS?
- Where did you eat *wild* CGS?
- Are they eaten more/only at special occasions? If Y, what kind of occasions?

1. Are CGS ever used in Traditional Chinese Medicine? Y/N/don’t know

If Y:

- What is their main medicinal use?

- Have you ever used CGS-based medicine? If Y, when?

1. Do people in this community have any other uses for *wild* CGS? Y/N/don’t know

If Y, describe these uses:

1. Has anybody (e.g. local people, reserve staff, farm staff, local authorities) ever released any CGS into any rivers in this region? Y/N/don’t know

If Y:

- How often does this happen?

- How many animals have been released?

- When was the most recent CGS release?

- Who releases the CGS?

- Where do these CGS come from?

- Where have they been released? (try to provide exact location(s))

- Why do they get released here?

1. Do you farm CGS? Y/N

If Y:

- Where do you obtain them from?

- How many do you have at the moment?

- What is the maximum number of CGS that you have ever kept?

- How long have you kept CGS for?

- Who do you sell these CGS to?

- Have you ever released CGS into the wild? Where and when?

1. Does anybody else in this community farm CGS? Y/N/don’t know

If Y:

- How many people (i.e. households)?

- How many CGS in total are farmed in this community? (approximate guess)

- Where do they obtain them from?

- Who are they sold to?

- How long have CGS been farmed in this community?

- Have they ever been released into the wild? Where and when?

**C. FRESHWATER RESOURCE USE QUESTIONS**

1. Do you go fishing? Y/N

If Y:

- How regularly do you go fishing? (how many times a week, month, or year)

- When do you go fishing? (day or night)

- Where do you go fishing? (give locations AND approx. distance from community – mark all locations on map if possible)

- Do you go fishing more at certain times of year? If so, which times of year?

- Which fish species do you try to catch? (list names)

- What fishing methods do you use? (list all methods) Which is your main fishing method?

- Do you use electro-fishing? If YES, how often do you use this method?

- Do you use poison? If YES, how often do you use this method?

- Do you use explosives? If YES, how often do you use this method?

If N:

- Did you use to go fishing? If YES, where did you use to go fishing, and when did you stop going fishing?

1. Have you ever tried to catch CGS from the wild? Y/N

If Y:

- How regularly do you try to catch CGS? (how many times a week, month, or year)

- When do you try to catch CGS? (day or night)

- Where do you try to catch CGS? (give locations AND approx. distance from community – mark all locations on map if possible)

- Do you try to catch CGS more at certain times of year? If so, which times of year?

- What methods do you use to catch CGS? (list all methods)

- Why do you catch CGS? If it is sold, who is it sold for, and for what price?

- How many CGS have you caught in the past year? Give dates and locations for each individual.

- How many CGS have you caught in the past five years? Give dates and locations for each individual if possible.

- Have you always tried to catch CGS? If N, when did you start trying to catch them, and why?

- Do you still try to catch CGS today? If N, when did you stop trying to catch them, and why?

1. Approximately what proportion of people in this community try to catch CGS?

- All
- Over 50%
- Less than 50%
- Very few
- None
- [Record exact number if given, as well as proportion]

1. What methods do people in this community use to try to catch CGS?
2. Has the level of CGS collecting carried out by local people changed over the past ten years? Y/N/don’t know

If Y:

- Has it increased or decreased over this time period, and by how much?

- Why has it changed?

1. What are the main localities where people try to catch CGS?
2. Do you collect other aquatic species (e.g. frogs, crabs, crayfish, shrimp, mussels) from the wild? Y/N

If Y:

- How regularly do you do this activity? (how many times a week, month, or year)

- When do you do this activity? (day or night)

- Where do you do this activity? (give locations AND approx. distance from community – mark all locations on map if possible)

- Do you do this activity more at certain times of year? If so, which times of year?

- Which species/kinds of animals do you try to catch? (list names)

- What methods do you use to collect these species? (list all methods)

- Why do you collect these species?

If N:

- Did you use to do this? If YES, where did you use to do this, and when did you stop?

1. Approximately what proportion of people in this community go fishing in nearby rivers?

- All

- Over 50%

- Less than 50%

- Very few

- None

1. What are the main areas around here where people go fishing?
2. Approximately what proportion of people in this community collect frogs or other aquatic species from nearby rivers?

- All

- Over 50%

- Less than 50%

- Very few

- None

1. What are the main areas around here where people collect other aquatic species?
2. Approximately what proportion of people in this community use electro-fishing?

- All

- Over 50%

- Less than 50%

- Very few

- None

- [Record exact number if given, as well as proportion]

1. Approximately what proportion of people in this community use poison fishing?

- All

- Over 50%

- Less than 50%

- Very few

- None

- [Record exact number if given, as well as proportion]

1. Approximately what proportion of people in this community use explosives for fishing?

- All

- Over 50%

- Less than 50%

- Very few

- None

- [Record exact number if given, as well as proportion]

1. Do people from other places ever visit the region near this community to collect CGS? Y/N/don’t know

If Y:

- How often does this happen?
- Does this tend to happen at a particular time of year? If Y, when?
- Do they try to collect adults, young or eggs?
- Where do these people come from?
- How many CGS have they caught over the past 12 months? (approx.)
- Why do they come here to collect CGS?

**D. CONSERVATION ATTITUDES QUESTIONS**

1. Do you think that any species in this region (aquatic and/or terrestrial) have declined over the past ten years? Y/N/don’t know

If Y, list all species the informant thinks has declined; describe the level of decline over the past ten years (approximate); describe what the informant thinks is responsible for this decline.

1. Have any species disappeared completely from this region during your lifetime? Y/N/don’t know

If Y, list all species, and the approximate date that these species disappeared from the local region.

*Also ask specifically about any known tiger sightings, and record all details (date, location) – this question can act as a control.*

1. Has the CGS population in this region stayed the same or changed at all (do NOT specifically ask if declined) during your lifetime? Same/changed/don’t know

If Y, describe the amount of change during this period (approximate); when it started to change; and describe what the informant thinks is responsible for this change.

1. What do you think the main threats are to the CGS population in this region? Please rank in order of importance, with 1=high importance. (*Read out the categories*)

- Overharvesting by local people
- Overharvesting by other people not from this community
- Water pollution
- Lack of food
- Water development projects (e.g. weirs, dams)
- Other (describe)
- No threats

1. What are your attitudes about whether CGS should be protected? (*Read out the categories*)

- They should be protected everywhere
- They should only be protected in nature reserves
- No need to protect them
- Other (describe)
- No opinion

1. Do you think that you or your community would get any benefit if CGS was protected? Y/N/don’t know

If YES, why would there be a benefit?

1. Are any particular aquatic species in this region specifically protected by law? Y/N/don’t know

If Y, list all species, and try to describe what this legislation is (i.e. whether they can/can’t hunt it, etc)

1. Is wild CGS specifically protected by law? Y/N/don’t know

If Y, try to describe what this legislation is (i.e. whether they can/can’t hunt it, etc)

1. Are any fishing methods banned in this region? Y/N/don’t know

If Y, ask the informant to name these methods.

1. Are any areas where fishing cannot be carried out? Y/N/don’t know

If Y, ask the informant to name these localities/rivers.

1. Have the nearby rivers changed at all during the past ten years? Y/N/don’t know

If Y, how have they changed in quality? (*NB: differentiate between water pollution and rubbish in the water if they say “pollution”.*) What has caused this change (e.g. water structures such as weirs or dams)?

1. Has anyone ever been prosecuted for illegal fishing around here? Y/N/don’t know

If Y, how often has this happened? Which village/town were they from? What charge/fine/sentence was there?

1. Has anyone ever been prosecuted for catching CGS around here? Y/N/don’t know

If Y, how often has this happened? Which village/town were they from? What charge/fine/sentence was there?

1. Has anyone ever been prosecuted for poaching/collecting any other animal or plant species around here? Y/N/don’t know

If Y, how often has this happened? Which village/town were they from? What charge/fine/sentence was there?

Space for further comments if necessary:

**Appendix S2 - Power Analysis**

We used our observed data structure (replication of records within and among grouping levels of random effects) to investigate our probability of detecting a real effect given our baseline probability in the reference category of model history, and a range of true differences in reporting probabilities. For all models, we incorporated overdispersion as observed in the true models, modelled as extra-Binomial variation on the link scale (Harrison 2015).

Given our data, we asked what the statistical power would be to detect a difference of x%, where x was varied from 2 to 20% in increments of 2. We simulated 1000 datasets per value of x and calculated the % of iterations where we could detect a significant difference between the two categories. For any difference of 10% of greater, we had >80% power to detect that difference given the structure of our data.

Our analysis of statistical power revealed that for our data structure, we would have >80% power to detect a difference of 10% or more in reporting probabilities.

**Table S1** Chinese giant salamander county-level sighting data.

| **County** | **Selection method** | **Total interviews** | **Total respondents seen salamander** | **Most recent reported sighting (yr ago)** | **Mean reported last sighting (yr ago)** |
| --- | --- | --- | --- | --- | --- |
| Jingde | historical record | 30 | 0 | − | − |
| Jixi | historical record | 30 | 0 | − | − |
| Liuan | habitat suitability model | 29 | 12 | 1 | 21.1666667 |
| Qimen | habitat suitability model | 30 | 2 | 4 | 20.5 |
| Xiuning | habitat suitability model | 30 | 28 | 0 | 22.3928571 |
| Yixian | historical record | 30 | 2 | − | − |
| Chengkou | habitat suitability model | 30 | 20 | 0 | 14.0526316 |
| Pengshui | habitat suitability model | 30 | 24 | 0 | 13.0952381 |
| Wushan | habitat suitability model | 30 | 27 | 6 | 26.4347826 |
| Pingnan | historical record | 30 | 1 | 3 | 3 |
| Cheng | habitat suitability model | 32 | 22 | 0 | 11.8333333 |
| Gaolan | habitat suitability model | 30 | 0 | − | − |
| Liangdang | habitat suitability model | 21 | 17 | 2 | 15.0588235 |
| Lintao | habitat suitability model | 30 | 0 | − | − |
| Liannan | habitat suitability model | 30 | 19 | 0 | 24.5294118 |
| Beiliu | habitat suitability model | 30 | 1 | 10 | 10 |
| Guiping | habitat suitability model | 31 | 9 | 0 | 21.7777778 |
| Jinxiu | habitat suitability model | 30 | 17 | 1 | 27.4375 |
| Longsheng | habitat suitability model | 31 | 7 | 2 | 21.5714 |
| Mengshan | habitat suitability model | 30 | 19 | 3 | 22.6842 |
| Napo | habitat suitability model | 33 | 2 | 0 | 35.5 |
| Rongshui | habitat suitability model | 30 | 21 | 0 | 24.4 |
| Tianlin | habitat suitability model | 30 | 6 | 7 | 21.8 |
| Xilin | habitat suitability model | 30 | 1 | 4 | 4 |
| Ziyuan | historical record | 31 | 5 | 20 | 30 |
| Cengong | historical record | 30 | 20 | 2 | 23.5384615 |
| Changshun | historical record | 30 | 0 | − | − |
| Danzhai | historical record | 30 | 19 | 0 | 23.6842105 |
| Dushan | historical record | 30 | 21 | 5 | 9.7 |
| Duyun | historical record | 30 | 21 | 12 | 26.8947368 |
| Fenggang | historical record | 30 | 19 | 1 | 13.1666667 |
| Fuquan | historical record | 30 | 24 | 4 | 13.4166667 |
| Guiding | historical record | 30 | 20 | 3 | 14.5294118 |
| Guiyanghuaxi | habitat suitability model | 30 | 7 | 20 | 34.75 |
| Huangping | historical record | 30 | 19 | 8 | 12.3846154 |
| Jiangkou | historical record | 30 | 30 | 0 | 13.0333333 |
| Jianhe | historical record | 30 | 20 | 1 | 28.5 |
| Jinping | historical record | 30 | 21 | 2 | 16.95 |
| Kaili | historical record | 30 | 25 | 1 | 9.42857143 |
| Kaiyang | historical record | 30 | 13 | 20 | 35.5 |
| Leishan | historical record | 30 | 6 | 1 | 1 |
| Liping | historical record | 30 | 0 | − | − |
| Longli | historical record | 30 | 22 | 4 | 18.8095 |
| Majiang | historical record | 30 | 19 | 2 | 14.6 |
| Meitan | historical record | 30 | 5 | 54 | 54 |
| Pingtang | habitat suitability model | 30 | 30 | 0 | 3.5625 |
| Sansui | historical record | 30 | 16 | 0 | 7.4545 |
| Shibing | historical record | 30 | 20 | 2 | 14.0526 |
| Songtao | habitat suitability model | 30 | 28 | 34 | 35.4285714 |
| Taijiang | historical record | 30 | 18 | 2 | 16.4444 |
| Tianzhu | historical record | 30 | 16 | 5 | 23.5333 |
| Wanshantequ | historical record | 30 | 30 | 1 | 15.2631579 |
| Wengan | historical record | 30 | 12 | 16 | 27.6666667 |
| Xifeng | historical record | 30 | 0 | − | − |
| Xiuwen | historical record | 30 | 15 | 29 | 37.3333333 |
| Yuqing | historical record | 30 | 22 | 0 | 0 |
| Zhenyuan | historical record | 30 | 14 | 10 | 18.5454546 |
| Zunyi | historical record | 30 | 23 | 2 | 12.6470588 |
| Jiaozuo | habitat suitability model | 36 | 2 | 28 | 28 |
| Jiyuan | habitat suitability model | 31 | 19 | 5 | 17.1428571 |
| Xinxian | habitat suitability model | 30 | 8 | 4 | 12.625 |
| Badong | habitat suitability model | 30 | 21 | 4 | 28.6190476 |
| Baokang | habitat suitability model | 32 | 27 | 1 | 23.125 |
| Danjiangkou | habitat suitability model | 30 | 27 | 4 | 26.0869565 |
| Macheng | habitat suitability model | 29 | 26 | 3 | 19.7692308 |
| Chaling | habitat suitability model | 30 | 0 | − | − |
| Chengbu | historical record | 11 | 6 | 4 | 33 |
| Guidong | historical record | 30 | 7 | 11 | 17.3333333 |
| Guzhang | historical record | 20 | 10 | 18 | 27.6 |
| Hengshan | habitat suitability model | 30 | 0 | − | − |
| Jiangyong | habitat suitability model | 30 | 3 | 20 | 35.3333333 |
| Longshan | historical record | 29 | 11 | 5 | 22 |
| Sangzhi | historical record | 30 | 28 | 0 | 12.28 |
| Suining | historical record | 30 | 0 | − | − |
| Xinhuangdong | historical record | 26 | 4 | 22 | 29.25 |
| Yongding | historical record | 30 | 28 | 0 | 23.7142857 |
| Yongshun | historical record | 30 | 22 | 3 | 33.631579 |
| Zhijiang | historical record | 27 | 5 | 30 | 40.25 |
| Jingan | habitat suitability model | 28 | 28 | 0 | 10.1428571 |
| Tonggu | historical record | 30 | 3 | 5 | 26.3333333 |
| Fengxian | habitat suitability model | 30 | 25 | 5 | 17.92 |
| Lueyang | habitat suitability model | 30 | 21 | 1 | 19 |
| Zhouzhi | habitat suitability model | 30 | 19 | 2 | 10.8947368 |
| Yuanqu | habitat suitability model | 30 | 0 | − | − |
| Baoxing | habitat suitability model | 30 | 2 | 13 | 33.5 |
| Emei | habitat suitability model | 30 | 17 | 0 | 16.8125 |
| Hejiang | habitat suitability model | 30 | 0 | − | − |
| Hongya | habitat suitability model | 30 | 24 | 5 | 31.0833333 |
| Jiuzhaigou | habitat suitability model | 30 | 3 | 0 | 5.33333333 |
| Mabian | habitat suitability model | 30 | 20 | 12 | 23.6 |
| Ningnan | habitat suitability model | 30 | 2 | 6 | 13 |
| Pingshan | habitat suitability model | 30 | 7 | 14 | 31.7142857 |
| Xingwen | habitat suitability model | 30 | 24 | 0 | 14.6956522 |
| Yiliang | habitat suitability model | 30 | 18 | 0 | 13 |
| Jingning | historical record | 30 | 12 | 16 | 22 |
| Longquan | habitat suitability model | 29 | 18 | 10 | 22 |
| Panan | historical record | 28 | 1 | 0 | 0 |
| Suichang | historical record | 29 | 12 | 0 | 33 |
